# Mini-LED, Micro-LED and OLED displays: present status and future perspectives

**DOI:** 10.1038/s41377-020-0341-9

**Published:** 2020-06-18

**Authors:** Yuge Huang, En-Lin Hsiang, Ming-Yang Deng, Shin-Tson Wu

**Affiliations:** College of Optics and Photonics, University of Central Florida, Orlando, FL 32816 USA

**Keywords:** Inorganic LEDs, Displays

## Abstract

Presently, liquid crystal displays (LCDs) and organic light-emitting diode (OLED) displays are two dominant flat panel display technologies. Recently, inorganic mini-LEDs (mLEDs) and micro-LEDs (μLEDs) have emerged by significantly enhancing the dynamic range of LCDs or as sunlight readable emissive displays. “mLED, OLED, or μLED: who wins?” is a heated debatable question. In this review, we conduct a comprehensive analysis on the material properties, device structures, and performance of mLED/μLED/OLED emissive displays and mLED backlit LCDs. We evaluate the power consumption and ambient contrast ratio of each display in depth and systematically compare the motion picture response time, dynamic range, and adaptability to flexible/transparent displays. The pros and cons of mLED, OLED, and μLED displays are analysed, and their future perspectives are discussed.

## Smaller LEDs usher in more advanced displays

Mini and micro light-emitting diodes (LEDs) could move to the centre-stage of display screen technologies once they mature. Shin-Tson Wu of the University of Central Florida and colleagues analysed the pros, cons, and future prospects of the latest display screen technologies, especially for use in smartphones, smart watches, virtual and augmented reality, and heads-up vehicle displays. These applications require bright, flexible, transparent, and power-efficient displays. The currently dominant liquid crystal displays (LCDs) require a backlight unit, dictating their shape and flexibility. LCDs with a backlight unit made from mini LEDs are becoming rapid contenders to the conventional technology. So are displays using organic light-emitting diodes, but these are limited in their brightness and lifespans. Emissive displays made from mini and micro-LEDs show huge potential once manufacturing costs can be brought down.

## Introduction

Display technology has become ubiquitous in our daily life; its widespread applications cover smartphones, tablets, desktop monitors, TVs, data projectors and augmented reality/virtual reality devices. The liquid crystal display (LCD) was invented in the late 1960s and early 1970s^1–4^. Since the 2000s, LCDs have gradually displaced bulky and heavy cathode ray tubes (CRTs) and have become the dominant technology^5,6^. However, an LCD is nonemissive and requires a backlight unit (BLU), which not only increases the panel thickness but also limits its flexibility and form factor. Meanwhile, after 30 years of intensive material^7–14^ and device development and heavy investment in advanced manufacturing technologies, organic light-emitting diode (OLED) displays^7,14–17^ have grown rapidly, enabling foldable smartphones and rollable TVs. In the past few years, emissive OLED displays have gained momentum and have competed fiercely with LCDs in TVs and smartphones because of their superior unprecedented dark state, thin profile, and freeform factor. However, some critical issues, such as burn-in and lifetime, still need to be improved. Recently, micro-LEDs (μLEDs)^18–27^ and mini-LEDs (mLEDs)^24,25,28^ have emerged as next-generation displays; the former is particularly attractive for transparent displays^19,29–31^ and high luminance displays^21–23^, while the latter can serve either as a locally dimmable backlight for high dynamic range (HDR) LCDs^24,28^ or as emissive displays^21–24^. Both mLEDs and μLEDs offer ultrahigh luminance and long lifetimes. These features are highly desirable for sunlight readable displays, such as smartphones, public information displays, and vehicle displays. Nevertheless, the largest challenges that remain are the mass transfer yield and defect repair, which will definitely affect the cost. “LCD, OLED or μLED: who wins?” has become a topic of heated debate^11^.

To compare different displays, the following are important performance metrics: (1) a HDR and a high ambient contrast ratio (ACR)^32^, (2) high resolution or a high resolution density for virtual reality to minimize the screen-door effect, (3) a wide colour gamut^33–35^, (4) a wide viewing angle and an unnoticeable angular colour shift^6,36–40^, (5) a fast motion picture response time (MPRT) to suppress image blur^41,42^, (6) low power consumption, which is particularly important for battery-powered mobile displays, (7) a thin profile, freeform, and lightweight system, and (8) low cost.

In this review paper, we compare the performance of mLEDs, OLEDs and μLEDs according to the abovementioned criteria. In particular, we evaluate the power consumption and ACR of each display in depth and systematically compare the dynamic range, MPRT, and adaptability to flexible and transparent displays. The pros and cons of mLED, μLED, and OLED displays are analysed, and their future perspectives are discussed.

## Device configurations

**Fig. 1:**
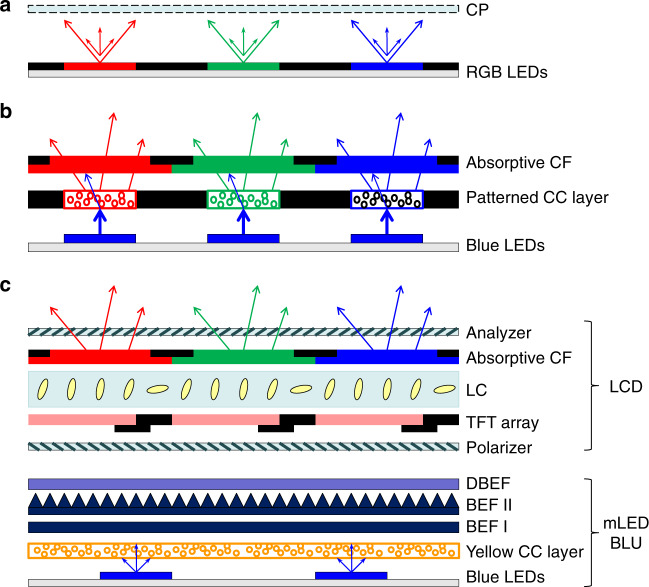
Display system configurations. **a** RGB-chip mLED/μLED/OLED emissive displays. **b** CC mLED/μLED/OLED emissive displays. **c** mini-LED backlit LCDs

Both mLED, μLED and OLED chips can be used as emissive displays, while mLEDs can also serve as a BLU for LCDs. Figure 1 illustrates three commonly used device configurations: red, green and blue (RGB)-chip emissive displays^26,27^ (Fig. 1a), colour conversion (CC) emissive displays^25^ (Fig. 1b), and mLED-backlit LCDs^24,28^ (Fig. 1c). In emissive displays (Fig. 1a, b), mLED/μLED/OLED chips serve as subpixels. In a nonemissive LCD (Fig. 1c), an mLED backlight is segmented into a zone structure; each zone contains several mLED chips to control the panel luminance, and each zone can be turned on and off selectively. The LC panel consists of M and N pixels, and each RGB subpixel, addressed independently by a thin-film transistor (TFT), regulates the luminance transmittance from the backlight. The full-colour images are generated differently in these three types. In Fig. 1a, RGB LED chips are adopted. Each LED will emit light in both the upward and downward directions. To utilize downward light, a reflective electrode is commonly deposited at the bottom of each LED chip. However, such a reflector also reflects the incident ambient light, which could degrade the ACR^32^. One solution is to adopt tiny chips to reduce the aperture ratio and cover the nonemitting area with a black matrix to absorb the incident ambient light^26^. This strategy works well for inorganic LEDs. However, for OLED displays, a large chip size helps to achieve a long lifetime and high luminance^43^. Under such conditions, to suppress the ambient light reflection from bottom electrodes, a circular polarizer (CP) is commonly laminated on top of the OLED panel to block the reflected ambient light from the bottom electrodes.

In Fig. 1b, each blue LED chip pumps a subpixel in the patterned CC layer (quantum dots or phosphors)^44^. An absorptive colour filter (CF) array is registered above to absorb unconverted blue light^44,45^ and suppress ambient excitations. This filter also enhances the ACR so that no CP is required. In some designs, a distributed Bragg reflector (DBR) is inserted to selectively recycle the unconverted blue light^46^ or to enhance the red/green output efficiency^47^. In Fig. 1c, blue mLED chips pump a yellow CC layer^48^ to generate white backlight. Additionally, a DBR could be optionally applied. In such a BLU, the mLED zones do not need to register with the subpixels so that a larger LED chip can be used. Because the CC layer scatters light, up to two brightness enhancement films (BEFs) can be employed to collimate light onto the on-axis direction. A dual brightness enhancement film (DBEF)^49^ can be inserted to transmit the preferred polarization, which is parallel to the transmission axis of the first polarizer and to recycle the orthogonal polarization. The transmitted light is modulated by the LCD with an absorptive CF array. In some designs, RGBW CFs instead of RGB CFs are employed to enhance the optical efficiency.

## Power consumption

The power consumption of mLED/μLED/OLED displays is primarily determined by the driving circuitry designs, LED quantum efficiency and optical system efficiency. In this section, we describe a power consumption evaluation model and give exemplary calculations on each display technology.

### Pulse amplitude modulation (PAM) driving schemes

**Fig. 2:**
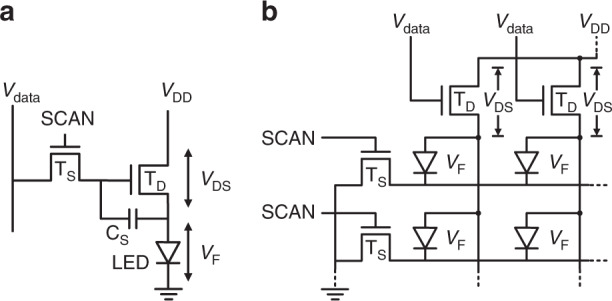
Pulse amplitude modulation LED driving schematics. **a** 2T1C active matrix and **b** basic passive matrix circuitries

PAM^50^, which is also called analogue driving, is commonly used in emissive OLED displays^51,52^. PAM is also an intuitive choice for μLED drivers. Both active matrix (AM) and passive matrix (PM) addressing techniques can be adopted in PAM^53^. Figure 2a shows a basic 2 transistors and 1 capacitor (2T1C) subpixel circuitry in AM addressing. In an emissive display panel with *M* by *N* pixels, the circuitry in Fig. 2a is arrayed by 3*M* columns (each pixel contains RGB subpixels) and *N* rows. T_S_ denotes the *switching* TFTs to sequentially turn on the LEDs, and T_D_ stands for the *driving* TFTs regulating the current flowing to the LEDs. For each row, T_S_ is only open for 1/*N* of the whole frame time (*T*_*f*_), during which the data voltage (*V*_data_) is loaded to the gate of T_D_, and then T_S_ is switched off. A storage capacitance (C_s_) holds the voltage so that T_D_ is kept open for the remainder of the frame time. Therefore, in AM addressing, the LED emits light for a *T*_*f*_. Figure 2b illustrates the arrayed PM driving circuitry. Here, no storage capacitance is employed. Thus, each LED only emits light for a short period (*T*_*f*_/*N*). To achieve the same effective luminance, the instant luminance in the PM should be *N* times higher than that of the AM.

### Power evaluation model of full-colour LED displays

**Fig. 3:**
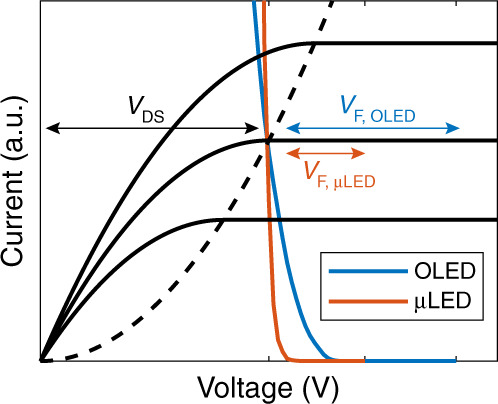
Operating spots of OLED displays and μLED displays. *V*_DS_: the TFT drain-to-source voltage. *V*_*F, OLED*_: the OLED forward voltage. *V*_*F, μLED*_: the μLED forward voltage

Our evaluation model is an improvement over those models reported by Lu^51^ and Zhou^52^. From the circuits in Fig. 2, the static power on each subpixel is mainly comprised of the LED’s power (*P*_LED_) and the driving TFT’s power (*P*_TFT_) as:

1 $$P_{\mathrm{static}} = P_{\mathrm{LED}} + P_{\mathrm{TFT}} = \left( {V_F + V_{\mathrm{DS}}} \right) \cdot I$$

where *I* is the current through the T_D_ and LED, *V*_*F*_ is the LED forward voltage, and *V*_DS_ is the drain-to-source voltage of the T_D_. In operation, the LEDs are current-driven devices, and T_D_ serves as a current source. The gate-to-source voltage (*V*_GS_) of the T_D_ controls *I*, and *I* determines the LED emittance. In the TFT part^50^, each solid black line in Fig. 3 denotes the *I*-*V*_DS_ curve at a given *V*_GS_. The dashed black lines delineate the border between the linear region (the left) and saturation region (the right). In the saturation region, *I* hardly changes with *V*_*DS*_ so that it is one-to-one mapped to *V*_GS_. Therefore, in designs, *V*_DS_ should exceed the following minimal value:

2 $$V_{\mathrm{DS}\_\min } = \sqrt {\frac{{2I}}{{\mu C_{ox}\frac{{W_T}}{{L_T}}}}} $$

in full brightness. In Eq. (2), we see that the region border (dashed lines in Fig. 3) is a function of carrier mobility (*μ*_*T*_), gate capacitance per unit area (*C*_ox_), channel width (*W*_*T*_) and channel length (*L*_*T*_).

Next, let us consider the LED part. The blue curve in Fig. 3 shows the OLED *I*-*V*_*F*_ characteristics with the flipped voltage. The intersection of the black dashed lines and the blue curve denotes the *I* and *V*_DS*_*min_ at full brightness. Then, the minimal required voltage across the T_D_ and LED is:

3 $$V_{\mathrm{DD}\_\min } = V_{\mathrm{DS}\_\min } + V_F$$

where *V*_DD_ is determined by the highest grey level and remains unchanged at lower grey levels. Taking an instance in Fig. 3, the operation current decreases from the highest grey level (the middle solid black curve) to a lower one (the lowest solid black curve). We can observe that the intersection of the blue curve and the solid black curve is right-shifted, indicating a decrease in *V*_*F*_ and an increase in *V*_DS_. The intersection point still dwells in the saturation region. The red curve in Fig. 3 depicts the *I*-*V*_*F*_ characteristics of the μLED. We can see that the behaviour of the μLED display is the same as that of the OLED display, except for a lower *V*_*F*_.

Notably, the *V*_*F*_ values of the μLED chip are lower than those of the OLED; this result is widely observed in the *J*-*V*_*F*_ characteristics. The relationship between the current density of μLED (*J*_μLED_) and *V*_*F*_ can be described by the Shockley model^54,55^:

4 $$J_{\upmu \mathrm{LED}} = J_s\left( {e^{V_F/nV_T} - 1} \right)$$

where *J*_*s*_, *V*_*T*_ and *n* stand for the saturation current density, the thermal voltage and the ideality factor, respectively. On the other hand, because of the small intrinsic charge density in organic materials, the current density of the OLED (*J*_OLED_) is space-charge limited^16,17,56^. According to the space-charge-limited-current (SCLC) model, the *J*-*V*_*F*_ characteristic of OLEDs follows the famous Mott-Gurney law^57^:

5 $$J_{\mathrm{OLED}} = \frac{9}{8}\varepsilon _0\varepsilon _r\mu \frac{{V_F^2}}{{d^3}}$$

Here, *ε*_*0*_ is the vacuum permittivity, *ε*_*r*_ is the relative permittivity of the OLED material, and *d* is the distance between the OLED electrodes. In Eq. (5), the free carrier mobility (*μ*) is a function of the electric field (*E* = *V*_*F*_/*d*)^58^:

6 $$\mu = \mu _0e^{0.89\beta \sqrt E }$$

where *μ*_0_ is the carrier mobility at a zero electric field and *β* is the Poole-Frenkel factor. Because of its much lower mobility, the OLED exhibits a higher threshold voltage and lower *J*-*V*_*F*_ curve slope than the μLED, leading to a higher operation voltage. Exemplary calculations are given in the Supplementary Information.

From Eq. (1), we find that the power consumption ratio between the TFT and LED is equal to *V*_DS_/*V*_*F*_. From Fig. 3, the high *V*_DS_/*V*_*F*_ ratio indicates that the TFT may not be an efficient driver for the mLED/μLED displays. In the experiment, we also confirmed that TFTs could consume more power than LED chips in an mLED/μLED display. Later, in this section, we will discuss how to reduce *P*_TFT_.

Apart from *P*_static_, the charge and discharge in *C*_*s*_ and the parasitic capacitance of data/scan lines in Fig. 2a generate the dynamic power consumption (*P*_dyn_)^55^. However, because *P*_dyn_ is much smaller than *P*_static_, the power evaluation in this part will only consider *P*_static_.

In a full-colour display, the driving voltage is determined by the following procedures: First, we determine *V*_*F*_ and *I* for each RGB chip according to LED L-I-V characteristics and panel specifications. Next, we adopt the proper TFT type and *W*_*T*_/*L*_*T*_ value to provide the required *I* with a reasonable *V*_DS_min_ (Eq. (2)) and *V*_DD_min_ (Eq. (3)). Last, because the *j* = R, G, B subpixels are integrated in a single panel, the common voltage (*V*_DD,*W*_) is

7 $$V_{\mathrm{DD},W} = \max (V_{\mathrm{DD}\_\min ,j})$$

**Fig. 4:**
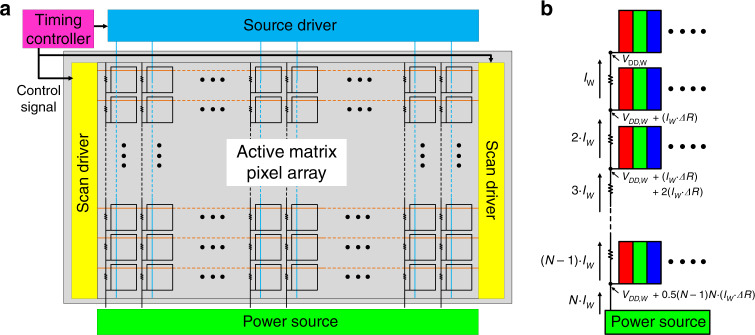
Illustration of *V*_DD_ voltage drop. **a** System schematic of an AM panel. **b** Voltage drop on a *V*_DD_ line

Apart from the power consumption on each subpixel, in AM panels, scan drivers and source drivers are employed for updating the driving current of the emissive device, as Fig. 4a shows. In addition, the wiring line has a parasitic resistor (Table 1). As shown in Fig. 4b, if *N* pixels are connected to one *V*_DD_ line in parallel, then the voltage across each pixel is reduced gradually from the power source to the pixel at the end^59^. Then, we can calculate the power loss on the parasitic resistor (*P*_resistor_) and on the voltage drop compensation (*P*_drop_) by

8 $$P_{\mathrm{resistor}} = \mathop {\sum}\limits_{i = 1}^{N - 1} {\left( {iI_W} \right)^2 \cdot \Delta R} = \frac{{\left( {N - 1} \right)N\left( {2N - 1} \right)}}{6} \cdot I_W^2 \cdot \Delta R$$

9 $$P_{\mathrm{drop}} = \mathop {\sum}\limits_{i = 1}^{N - 1} {\frac{{i\left( {i + 1} \right)}}{2}} \cdot I_W^2 \cdot \Delta R = \frac{{\left( {N - 1} \right)N\left( {N + 1} \right)}}{6} \cdot I_W^2 \cdot \Delta R$$

10 $$P_{\mathrm{resistor}} + P_{\mathrm{drop}} \approx \frac{{N^3}}{2} \cdot I_W^2 \cdot \Delta R$$

**Table 1:** RGB chromaticity coordinates of the reported mLED/μLED/OLED displays in comparison with Rec. 2020 in CIE 1931

	(x_R_, y_R_)	(x_G_, y_G_)	(x_B_, y_B_)	Colour gamut (Rec. 2020)
Rec. 2020^33,34^	(0.708, 0.292)	(0.170, 0.797)	(0.131, 0.046)	100%
RGB OLED^72^	(0.706, 0.294)	(0.188, 0.757)	(0.136, 0.052)	91.8%
RGB μLED^100^	(0.701, 0.300)	(0.168, 0.754)	(0.135, 0.056)	91.4%
CC μLED^101^	(0.698, 0.302)	(0.169, 0.766)	(0.134, 0.051)	93.1%
mLED-LCD^104^	(0.706, 0.294)	(0.158, 0.792)	(0.134, 0.048)	95.8%

Here, *I*_*W*_ is the current for each full-colour pixel, and Δ*R* is the *V*_DD_ line resistance across a pixel pitch. It is worth pointing out that although the previous model mentioned a voltage drop^51,52^, *P*_drop_ was not considered in the calculation. To reduce these power losses, the *N* rows in the panel may be segmented into *N*_*g*_ groups with independent *V*_DD_ transmission. Then, *P*_resistor_ and *P*_drop_ can be reduced to 1/*N*_*g*_^2^. Considering the whole panel, the total power loss caused by the wire resistor (*P*_wire_) is

11 $$P_{\mathrm{wire}} \approx \frac{{N^3 \cdot M}}{{2N_g^2}} \cdot I_W^2 \cdot \Delta R$$

From Eqs. (1), (3), (7) and (11), the total power consumption of a full-colour display is

12 $$\begin{array}{c}P_{\mathrm{total}} \approx \left( {P_{\mathrm{LED}} + P_{\mathrm{TFT}}} \right)_{\mathrm{RGB}} \;+\;P_{\mathrm{wire}}\\ \approx V_{\mathrm{DD},W} \cdot I_W \cdot N \cdot M + \frac{{N^3 \cdot M}}{{2N_g^2}} \cdot I_W^2 \cdot \Delta R\end{array}$$

### Power efficacy under PAM and improvement strategies

The wall-plug efficiency (WPE [unit: W/W]) reflects an LED’s power efficiency, which is the output optical power (*P*_op_) over the input electrical power (*P*_LED_):

13 $${\mathrm{WPE}} = \frac{{P_{\mathrm{op}}}}{{P_{\mathrm{LED}}}} = \frac{{E_{\mathrm{ph}} \cdot \mathrm{EQE}_{\mathrm{chip}}}}{{e \cdot V_F}}$$

In Eq. (13), *E*_ph_, EQE_chip_ and *e* represent the photon energy, LED external quantum efficiency (EQE) and elementary charge, respectively. The luminous flux from an LED (Φ_LED_ [unit: lm]) is related to *P*_op_ and luminous efficacy (*K*) as:

14 $${\Phi}_{\mathrm{LED}} = K \cdot P_{\mathrm{op}}$$

15 $$K = \frac{{{\int} {V\left( \lambda \right)S\left( \lambda \right)d\lambda } }}{{{\int} {S\left( \lambda \right)d\lambda } }}$$

where *V*(*λ*) is the spectral luminous efficacy and *S*(*λ*) is the emission spectrum.

From Eqs. (13)–(15) and Eq. (1), the LED efficacy (*η*_LED_ [unit: lm/W]) and the circuit power efficacy (*η*_*p*_ [unit: lm/W]) can be expressed as ^60^:

16 $$\eta _{\mathrm{LED}} = \frac{{{\Phi}_{\mathrm{LED}}}}{{P_{\mathrm{LED}}}} = \frac{{K \cdot E_{\mathrm{ph}}}}{e} \cdot \frac{{\mathrm{EQE}_{\mathrm{chip}}}}{{V_F}}$$

17 $$\eta _p = \frac{{{\Phi}_{\mathrm{LED}}}}{{P_{\mathrm{static}}}} = \frac{{{\Phi}_{\mathrm{LED}}}}{{P_{\mathrm{LED}} \cdot \frac{{V_F + V_{\mathrm{DS}}}}{{V_F}}}} = \frac{{K \cdot E_{\mathrm{ph}}}}{e} \cdot \frac{{\mathrm{EQE}_{\mathrm{chip}}}}{{V_F + V_{\mathrm{DS}}}}$$

There are several methods to improve the power efficacy of mLED/μLED/OLED displays. For a lower *P*_wire_, we can segment the panel into more units (Eq. (11)) and employ low resistivity wire materials. For *P*_TFT_ and *P*_LED_, we discuss them as follows.

#### (a) P_TFT_ reduction on driving transistors

The *P*_TFT_ can be reduced by optimizing the T_D_ parameters. From Eqs. (1) and (2), higher *μ*_*T*_, higher *C*_ox_ and higher *W*_*T*_/*L*_*T*_ help lower *V*_DS_min_ and *P*_TFT_. Among them, *W*_*T*_ and *L*_*T*_ are circuit design parameters but should be adjusted in a reasonable range. In a high ppi (pixel per inch) display, the small area in each subpixel may not leave much space for a large-channel width (*W*_*T*_) TFT, especially when compensation circuits^61,62^ are needed. When the channel length (*L*_*T*_) is too short, electricity leakage becomes severe and causes a short-channel effect^55^. In addition, *V*_DS_ should be large enough to achieve 8-bit driving, even 10-bit or 12-bit driving for HDR displays.

On the other hand, *μ*_*T*_ and *C*_ox_ are TFT process parameters. The oxide layer at the TFT gate is designed to be properly thin to reach a balance between high *C*_ox_ and good insulation. High *μ*_*T*_ can be obtained from complementary metal-oxide-semiconductor (CMOS) transistors. Consequently, industry leaders began to substitute TFTs with CMOS driver integrated circuits (ICs)^22,23,26,63,64^: (a) In the PM addressing scheme, a few ICs function as many TFTs^29^. However, the resolution and size of PM displays are limited. Therefore, multiple PM blocks need to be tiled to obtain high-resolution and large-size displays. The major challenges of tiling designs are seam visibility and uniformity, which require small emission aperture and post-manufacturing calibrations, respectively^26^. (b) In the AM addressing scheme (Fig. 2a), each pixel has a unit circuit, and compensation designs are normally needed^61,62^. This scheme is space demanding and especially unfriendly to high ppi displays. The highly integrated IC mitigates this issue and provides more accurate current control in PAM. Moreover, this technology enables miniaturized pulse width modulation (PWM) driving circuits^26,29,55,65^. In 2015, Lumiode reported a transfer-free method to integrate silicon TFTs on AM μLED microdisplays^21^. In 2017, X-Celeprint demonstrated an AM μLED display with pixelated microscale ICs by microtransfer printing^29^. In 2018, JDC introduced a 2000-ppi μLED on a silicon backplane^65^. In 2019, LETI proposed fabricating elementary pixel units at the wafer scale and transferring them to a receiving substrate. In LETI’s design, each unit contains an RGB μLED set on a CMOS driving circuit^64^. Sony adopted a pixelated micro-IC in Crystal LED—their commercial tiling μLED display system^26^. The main drawback of IC drivers is that they have a higher cost than TFTs. As the number of employed ICs increases, the panel cost increases. Therefore, it is more cost-friendly to employ ICs in low-resolution BLUs than in high-resolution emissive displays.

#### P_LED_ reduction by high EQE_chip_*/V*_*F*_ operation

**Fig. 5:**
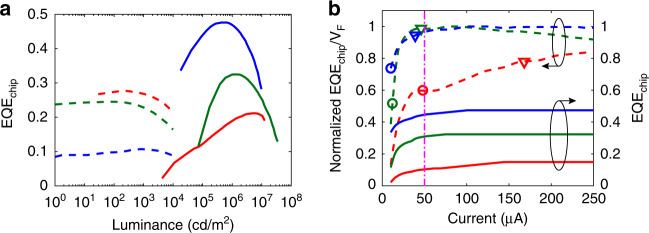
OLEDs and μLED characteristics. **a** EQE_chip_ as a function of chip luminance. The RGB dashed lines are for RGB OLEDs. The RGB solid lines are for RGB mLEDs. **b** Current-dependent EQE_chip_ (solid lines) and normalized EQE_chip_*/V*_*F*_ (dashed lines) of RGB mLEDs, as denoted by RGB colours, respectively

From Eq. (16), we find that *η*_LED_ is proportional to EQE_chip_/*V*_*F*_, indicating a high EQE_chip_/*V*_*F*_ operation preference. First, let us consider the EQE_chip_ characteristics (Fig. 5a). The RGB colour lines correspond to RGB colour chips. The *x*-axis is colour luminance. For instance, 1000 cd/m^2^ white light is mixed by approximately [R: 300 cd/m^2^, G: 600 cd/m^2^, B: 100 cd/m^2^] colour luminance. As the dashed lines show in Fig. 5a, the EQE_chip_ of the OLED^11,12,66^ remains flat in the normal operation range (<4000 cd/m^2^ mixed white light) but rolls off gradually as the luminance increases. On the other hand, the EQE_chip_ of 90 μm × 130 μm mLED chips (the solid lines in Fig. 5a) varies significantly with the luminance. The peak EQE_chip_ of the GB mLED/μLED chips is higher than that of the OLED but resides in the high luminance region. Here, we plot the chip luminance under constant illumination. In practical applications, designers may adopt a low aperture ratio (AP = 1~ 20%)^26^ and a low duty ratio (DR ~ 10%)^41,42^; under such conditions, the display luminance declines by a factor of (AP*·* DR), which is 2 ~ 3 orders lower than the original chip luminance. Optical films may further reduce the display luminance, which will be discussed later for each system configuration. It is worth mentioning that the EQE_chip_ of mLED/μLED is chip size-dependent. Although a very high EQE_chip_ (>80% for blue) has been achieved on large chip sizes^60,67^, for μLEDs (chip size < 50 μm), their EQE_chip_ is significantly reduced due to sidewall emission^27,68,69^ and insufficient light extraction^70^. We will discuss the size effect in the “Ambient contrast ratio” section. Overall, OLEDs exhibit higher EQE_chip_ than mLEDs/μLEDs with respect to red, green and white colours in the high aperture ratio and high DR designs at normal operation range (<4000 cd/m^2^ mixed white light).

The strong variation in EQE_chip_ makes operation spot optimization critical for mLED/μLED displays. Therefore, we plot the current-dependent EQE_chip_ and EQE_chip_/*V*_*F*_ in Fig. 5b. Taking AP = 2.5% and DR = 100% under AM PAM as an example, the mLED operation range is from *I* = 0 to the spots marked by circles to achieve 1500-cd/m^2^ peak luminance. In this range, the low EQE_chip_/*V*_*F*_ implies a low *η*_LED_. We may apply a low DR to shift the operation spots to a high EQE_chip_/*V*_*F*_ region and enhance the *η*_LED_. For instance, if DR = 20%, then the instant luminance should be increased by 5× to maintain the same average luminance. Then, the full-brightness driving spots are shifted to the triangles in Fig. 5b, corresponding to an EQE_chip_/*V*_*F*_ improvement of [30%, 91%, 28%] for the [R, G, B] chips, respectively. An alternative method is to constantly drive LEDs at high EQE_chip_/*V*_*F*_ spots under PWM^26,29,65^. As an example, at *I* = 50 μA (marked by the magenta dashed lines in Fig. 5b), the EQE_chip_/*V*_*F*_ of blue and green mLEDs increases by 31 and 91% from the circle spots, respectively. A higher EQE_chip_/*V*_*F*_ can be obtained at a higher current on the red chip, but the burdens on circuit electronics will be more demanding. Furthermore, hybrid driving^29,71^ is a method combining PAM and PWM, which enables both high bit depth and high efficiency.

### On-axis power efficacy in optical systems under PWM

We have discussed the power efficacy of full-colour LED panels. Considering the display system’s optical efficiency (*T*_sys_, which could be different for *j* = R, G, B subpixels), the ratio between luminous flux output from a subpixel (Φ [unit: lm]) and that emitted from the registered LED (Φ_LED_ [unit: lm]) is

18 $$\frac{{{\Phi}_j}}{{{\Phi}_{\mathrm{LED},j}}} = T_{\mathrm{sys},j}$$

In the CC type, if the blue light is converted to red and green with efficiency EQE_CC_, then on the *j* = R, G subpixels, Eq. (18) is modified as

19 $$\frac{{{\Phi}_j}}{{{\Phi}_{\mathrm{LED},B}}} = \frac{{K_j \cdot E_{\mathrm{ph},j}}}{{K_B \cdot E_{\mathrm{ph},B}}} \cdot \mathrm{EQE}_{\mathrm{CC},j} \cdot T_{\mathrm{sys},j}$$

Taking the aperture ratio and DR into account, the display luminance becomes [AP· DR· Φ/Φ_LED_] times the chip luminance. From Eqs. (16)–(19), the on-axis luminous power efficacy (*η* [unit: cd/W]) for *j* = R, G, B colours is

20 $$\eta _j = \frac{{L_j \cdot A_{\mathrm{pix}}}}{{P_j}} = \frac{{{\Phi}_j}}{{P_j \cdot F_j}} = \frac{{K_j \cdot E_{\mathrm{ph},j}}}{e} \cdot \frac{{\mathrm{EQE}_j \cdot T_{\mathrm{sys},j}}}{{V_j \cdot F_j}}$$

where *A*_pix_ is the pixel area and *F* [unit: sr] is the conversion coefficient from the on-axis luminous intensity [unit: cd] to the luminous flux Φ [unit: lm]. For emissive mLED displays, the LED’s angular emission profile is close to Lambertian, corresponding to *F* = π sr. The sidewall emission increases the ratio of light emitted to large angles^70^, leading to a larger *F*, which lowers the ratio of light contributing to the on-axis intensity. This effect is more severe on the smaller-sized μLEDs. The case is different in the BLU. BEFs and DBEFs are commonly used in BLUs to redistribute more light towards the normal direction with preferred polarization. As an example, *F* can be reduced to 0.96 sr by applying two BEFs and one DBEF (3M Vikuiti^TM^)^49^. To obtain D65 white light, the monochromatic luminance *L*_*j*_ is mixed in colour mixing ratio *r*_*j*_ by

21 $$L_j = L_W \cdot r_j$$

From Eqs. (20) and (21), the on-axis luminous power efficacy for mixed white light is

22 $$\eta _W = \frac{{L_W \cdot A_{\mathrm{pix}}}}{{\mathop {\sum}\limits_{j = R,G,B} {P_j} }} = \frac{{L_W \cdot A_{\mathrm{pix}}}}{{\mathop {\sum}\limits_{j = R,G,B} {\frac{{L_j \cdot A_{\mathrm{pix}}}}{{\eta _j}}} }} = \frac{1}{{\mathop {\sum}\limits_{j = R,G,B} {\frac{{r_j}}{{\eta _j}}} }}$$

To be noticed in Eqs. (20) and (22), in the evaluation of LED efficacy, *P*_*j*_ and *V*_*j*_ stand for *P*_LED*,j*_ and *V*_*F,j*_, respectively. On the other hand, in the analysis of circuit power efficacy, *P*_*j*_ and *V*_*j*_ mean *P*_static*,j*_ and *V*_DD*_W*_, respectively. Since *P*_TFT_ can be optimized by driving schemes, in the following discussions, we focus on the output LED efficacy. As discussed in Fig. 5b, we also assume that PWM is adopted so that LEDs work at the high EQE_chip_/*V*_*F*_ spot at *I* = 50 μA. In the following discussion, we evaluate the *η*_*W*_ of each display technology, and some exemplary calculation data are summarized in Tables S1–S4 in the Supplementary Information.

#### (a) RGB-chip emissive displays

In Fig. 1a, RGB chips are employed. A CP is laminated on large-aperture mLED/μLED/OLED displays, corresponding to *T*_sys_ = *T*_CP_ = 42%. Then, we modify Eq. (20) for the RGB-chip emissive displays as:

23 $$\eta _{\mathrm{RGB},j} = \frac{{K_j \cdot E_{\mathrm{ph}.j}}}{e} \cdot \frac{{\mathrm{EQE}_{\mathrm{chip},j} \cdot T_{\mathrm{CP},j}}}{{V_j \cdot F_j}}$$

After some algebra, we find that *η*_RGB*,W*_ of the mLED emissive displays is 6.8 cd/W (Table S1). More than half of the power is consumed by the red mLED due to its relatively low EQE_chip*,R*_. As shown in Fig. 5b, EQE_chip*,R*_ is more than 3× lower than EQE_chip*,B*_ and EQE_chip*,G*_ at 50 μA. The low EQE_chip*,R*_ originates from the low light extraction efficiency, since the red semiconductor material (AlGaInP) has a higher refractive index than the blue/green semiconductor material (InGaN)^70^. Technology innovation to improve EQE_chip*,R*_ of mLED is urgently needed. As the chip size shrinks to <50 μm (μLED), the peak EQE_chip_ decreases^27,68,69^. Later, in the “Ambient contrast ratio” section, we will show that *η*_*W*_ drops with reduced size, but ACR may increase.

For OLED displays, the evaluated *η*_RGB*,W*_ is 3.9 cd/W (Table S2) with EQE_chip_ = [0.27, 0.24, 0.10] for [R, G, B] colours^11,12,66,72^. A higher OLED EQE_chip_ has been achieved in labs with advancements in emitting mechanisms^10,14^, materials^10,14^, emitter orientation control^13^ and light extraction patterning^73^. However, the compromised lifetime, colour purity and production yield limit their commercial use. Overall, the higher *η*_RGB*,W*_ of the mLED than that of the OLED comes from the higher EQE_chip_ of the mLED. Compared with OLED materials, the robustness of inorganic LED materials facilitates light extraction patterning. It is also worth mentioning that OLED’s lowest EQE_chip_ falls on blue, but in inorganic LEDs, it is the red colour, as Fig. 5a demonstrates.

#### (b) Colour conversion emissive displays

As Fig. 1b depicts, the red/green colours are converted from blue LED chips, which bypasses the need for high EQE_chip_ red mLEDs/μLEDs. However, OLED displays rely on blue chips, which have lower efficiency and shorter lifetimes. In Fig. 1b, the patterned CC film is normally a quantum dot colour filter (QDCF)^44^. The overall EQE becomes a product of the blue chip EQE (EQE_chip*,B*_) and QDCF’s CC efficiency (EQE_QDCF_). Above that, the absorptive CF could be presented by its transmittance (*T*_CF_). Under such conditions, Eq. (20) is modified to:

24 $$\eta _{\mathrm{CC},j} = \frac{{K_j \cdot E_{\mathrm{ph}.j}}}{e} \cdot \frac{{\mathrm{EQE}_{\mathrm{chip},B} \cdot \mathrm{EQE}_{\mathrm{QDCF},j} \cdot T_{\mathrm{CF},j}}}{{V_B \cdot F_j}}$$

Using the same mLED chips, the *η*_*W*_ of the CC type (12.0 cd/W from Table S3) is ~1.8× higher than that of the RGB chip type (6.8 cd/W). This increase is mainly because *T*_*CF*_ ( = 0.7~ 0.9, depending on the RGB colours) is higher than *T*_CP_ ( = 0.42). If the aperture ratio of the mLED or μLED is small, then *η*_RGB*,W*_ can be doubled by removing the CP. Under such conditions, the *η*_*W*_ of the RGB-chip type and CC type are comparable. We will address this issue later in the “Ambient contrast ratio” section. In the above calculation, we used EQE_QDCF_ = 0.3 ~ 0.38 as reported by Nanosys^44^. If the EQE_QDCF_ can be further improved, then more power savings of the CC type can be realized.

#### (c) Mini-LED backlit LCDs

The main power consumption of the mLED-LCD originates from the BLU. In Fig. 1c, the blue LED light is converted to white through a yellow CC film with an efficiency EQE_QDEF_ ≈ 0.73^48^. Some optical films, such as DBR, diffuser, BEF and DBEF, may be added to the BLU, corresponding to a luminous transmission *T*_BLU_ ≈ 0.9. Then, the light is modulated by an LC panel whose optical efficiency *T*_LCD_ ≈ 5% for RGB CFs. The output on-axis power efficacy is

25 $$\eta _{\mathrm{LCD},j} = \frac{{K_j \cdot E_{\mathrm{ph}.j}}}{e} \cdot \frac{{\mathrm{EQE}_{\mathrm{chip},B} \cdot \mathrm{EQE}_{\mathrm{QDEF},j} \cdot T_{\mathrm{BLU}} \cdot T_{\mathrm{LCD}}}}{{V_B \cdot F_j}}$$

From Eq. (25), the calculated *η*_LCD*,W*_ is 4.1 cd/W (Table S4). Using this number, the power consumption of a 65-inch 4 K TV with 1000-cd/m^2^ peak luminance is *P*_LED*,W*_ = 284 W, which agrees very well with the measured 280 W. From the *η*_*W*_ viewpoint, mLED-LCDs have similar power consumption to RGB-chip OLED displays (*η*_RGB*,W*_ = 3.9 cd/W). These displays are approximately 3× lower than CC-based emissive mLED/μLED displays and CP-free RGB-chip emissive mLED/μLED displays. This ratio can be changed by other influencing factors: (1) Higher optical efficiency can be obtained with mLED-LCDs with RGBW CFs. (2) Compared with emissive displays, larger LEDs can be used in BLUs, enabling a higher EQE_chip_^27,68,69^ and a higher light extraction efficiency^70^. (3) *P*_TFT_ can be comparable or even larger than *P*_LED_ in TFT-driven emissive displays. (4) Under PAM, the *η*_LED_ is low if operated in the low current region for an emissive display, while a high EQE_chip_/*V*_*F*_ can be easily maintained in an mLED BLU.

## Contrast ratio and ACR

### Contrast ratio

The CR of an emissive display is inherently high. In a nonemissive LCD, its CR is limited by the depolarization effect mainly from the employed LC material, surface alignment and CFs^74,75^. Normally, the CR of an LCD is approximately 5000:1, 2000:1 and 1000:1 for the multidomain vertical alignment (MVA) mode^36^, fringe-field switching (FFS) mode^37^ and twisted-nematic (TN) mode^2^, respectively. To further enhance the CR, local dimming technology can be applied to reduce light leakage in the dark state^28,76–79^. A local dimming display system consists of dual modulation units, i.e., a segmented low-resolution mLED backlight and a high-resolution LCD panel. As discussed previously, this pre-modulation can be realized by a 2D arrayed mLED BLU. With a proper number of local dimming zones, the troublesome halo effect and clipping effect can be suppressed to an unnoticeable level^28,79^. Another method is to cascade two LCD panels^80–82^: a black-and-white low-resolution panel (e.g., 2K1K) to provide a local dimming effect and a high-resolution (8K4K) full-colour panel. Unlike an mLED backlight that can provide thousands of zones, such a dual-panel LCD can offer millions of zones at a fairly low cost, but the traded-off is the increased thickness.

### Ambient contrast ratio

In practical applications, the reflected ambient light (either from the external surface or from internal electrodes) is also perceived in addition to the displayed contents. The ACR is defined as^24,32^

26 $${\mathrm{ACR}} = \frac{{L_{\mathrm{on}} + \frac{{I_{\mathrm{am}}}}{\pi } \cdot R_L}}{{L_{\mathrm{off}} + \frac{{I_{\mathrm{am}}}}{\pi } \cdot R_L}} \approx 1 + \frac{{\pi \cdot L_{\mathrm{on}}}}{{I_{\mathrm{am}} \cdot R_L}}$$

Here, *L*_on_ and *L*_off_ (« *L*_on_ for high CR displays) are the on- and off-state luminance of the display, and *I*_am_ and *R*_*L*_ stand for the ambient illuminance and luminous reflection of display panel, respectively. From Eq. (26), a high *L*_on_ and a low *R*_*L*_ help to enhance the ACR. *L*_on_ can be boosted by the input power. *R*_*L*_ is related to the optical structure^24^ and can be suppressed by several approaches, such as anti-reflection coating on the substrates, the CP in RGB-chip emissive displays (Fig. 1a), the CF in CC emissive displays (Fig. 1b), and the crossed polarizers in mLED-LCDs (Fig. 1c). These methods can considerably suppress LED ambient reflection and QD ambient excitation. In these structures, *R*_*L*_ is mainly determined by the surface reflection (0.5 ~ 4%) rather than the emission aperture (AP), so it remains at a low level. To achieve high *L*_on_, the CP in RGB-chip emissive μLED displays can be removed to acquire doubled optical efficiency. In this design, due to the high LED reflectance, *R*_*L*_ substantially increases as *AP* increases. Therefore, a small chip size helps to enhance the ACR. The drawback of this small-chip strategy is the increased surface-to-volume ratio and the aggravated EQE loss from Shockley-Read-Hall non-radiative recombination^27,68,69^. Therefore, the LED chip size should be carefully chosen while balancing the optical reflectance with electrical power efficiency^24^. The optical structure that governs *R*_*L*_ and the chip size-dependent peak EQE are summarized in the Supplementary Information.

**Fig. 6:**
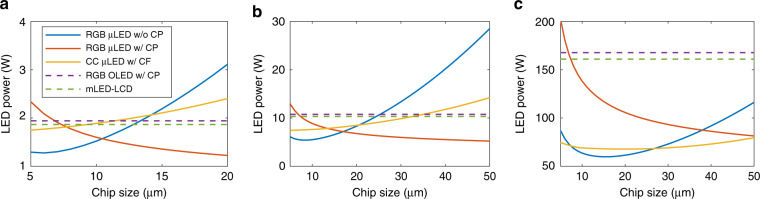
Chip size-dependent LED power consumption with different display technologies. **a** 50-μm pitch smartphone under 1500-lux overcast daylight for ACR=40:1. **b** 90-μm pitch notebook under 500-lux office light for ACR=100:1. **c** 375-μm pitch (65-inch 4K) TV under 150-lux living room ambient for ACR = 1000:1

Because displays with the same *L*_on_ can exhibit different ACRs^32^, when evaluating the efficiency, it would be more fair to compare the power consumption at the same human-perceived ACR rather than to reach the same luminance. With this motivation, we plot the ACR-determined power consumption in Fig. 6. Here, a smartphone (Fig. 6a), a notebook (Fig. 6b) and a TV (Fig. 6c) in full brightness under their corresponding viewing conditions are taken as examples. The LED power consumption is calculated by *L*_on_/*η*_*W*_ according to the power consumption section. In each application, five display structures are evaluated. For the CP-laminated RGB-chip mLED/μLED/OLED emissive displays (red curves and purple curves), *R*_*L*_ does not change with AP. As the chip size increases, the peak EQE_chip_ of the μLED increases, leading to a decreased power, as shown by the red curves. However, the size effect for RGB OLED displays (purple curves) is negligible. On the other hand, for the CP-free μLED emissive displays (blue curves and yellow curves), *R*_*L*_ increases with a larger AP. As chip size increases, both *R*_*L*_ and EQE_chip_ increase, but they have opposite effects on the ACR. As a result, the required LED power decreases first and then increases. This trend is more obvious for the RGB-chip type (blue curves) than for the CC type (yellow curves). This result is because the LED reflectance in the RGB-chip type is strong, while the CF array in the CC-based μLED emissive displays partially suppresses ambient excitations. For the applications shown in Fig. 6, the most power-efficient chip size is located at <20 μm. We also add mLED-LCDs (green curves) for comparison, although the actual chip size of the mLED (~200 μm) in the BLU is beyond the horizontal scale plotted in Fig. 6.

Based on Fig. 6, we find that for portable devices (Fig. 6a, b), the most power efficient choice is the RGB-chip μLED display. Both the small-chip CP-free design (blue curves) and large-chip CP-laminated structure (red curves) are outstanding. The intersection point of with/without-CP designs can be calculated by the following method. For a given display, the ACR of with/without-CP designs are shown as follows:

27 $$\begin{array}{l}\mathrm{ACR}_{\mathrm{CP}} = 1 + \frac{{\pi \cdot L_{\mathrm{on},\mathrm{CP}}}}{{I_{\mathrm{am}} \cdot R_{L,\mathrm{CP}}}}\\ \mathrm{ACR}_{\mathrm{no} - \mathrm{CP}} = 1 + \frac{{\pi \cdot L_{\mathrm{on},\mathrm{no} - \mathrm{CP}}}}{{I_{\mathrm{am}} \cdot R_{L,\mathrm{no} - \mathrm{CP}}}}\end{array}$$

The same power consumption to achieve the same ACR dwells at

28 $$\frac{{R_{L,\mathrm{CP}}}}{{R_{L,\mathrm{no} - \mathrm{CP}}}} = \frac{{L_{\mathrm{on},\mathrm{CP}}}}{{L_{\mathrm{on},\mathrm{no} - \mathrm{CP}}}} = T_{\mathrm{CP}}$$

For example, from Fig. 6a, the intersection of the blue and red curves occurs at 10.23 µm. At this critical chip size, the device reflectance ratio is *R*_*L*,CP_/*R*_*L*,no-PC_ = 0.04/0.095 = 0.42. For this 50-µm pitch smartphone, we suggest using RGB-chip μLED emissive displays, either with CP on a larger chip size (red curve) or without CP on a smaller chip size (blue curve). On the other hand, for long-pitch TV devices (Fig. 6c), the CP-free RGB-chip μLED emissive display (blue curve) still shows an advantage over the colour-converted display (yellow) on small chips (7–27 µm). However, the CC-type μLED is friendly to 30 ~ 50 µm chips; in this range, the fabrication technologies are more mature, and the manufacturing yield is higher.

## Response time and MPRT

The response time of mLED/μLED/OLED chips is several orders faster than that of LCs. However, we cannot conclude that mLED/μLED/OLED emissive displays provide a much smoother visual experience than LCDs. A widely used metric for the visual response time is MPRT^41,42^. MPRT is jointly determined by pixel response time (*τ*) and frame rate (*f* = 1/*T*_*f*_), and it can be calculated by a simplified equation proposed by Peng et al.^42^:

29 $${\mathrm{MPRT}} = \sqrt {\tau ^2 + \left( {0.8T_f} \right)^2} $$

**Fig. 7:**
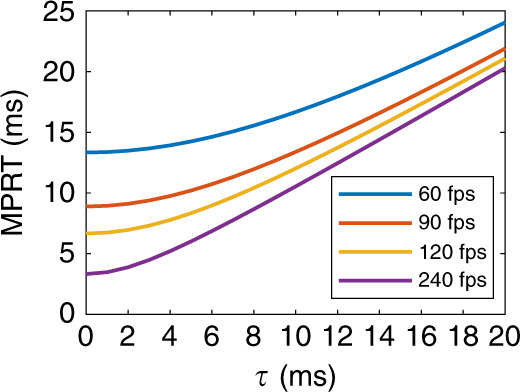
Pixel response time-dependent MPRT at different frame rates. fps: frames per second

From Eq. (29), a relatively long *τ* would slow down the MPRT. However, when *τ* « *T*_*f*_, MPRT is mainly determined by *T*_*f*_, so a high frame rate helps to reduce the MPRT. Figure 7 shows the simulated MPRT at four frame rates. For instance, at *f* = 60 fps, the MPRT of a 2-ms-response LCD is 13.5 ms, which is comparable with the μs-/ns-response emissive displays (MPRT = 13.3 ms). As the frame rate increases to 120 fps, the MPRT is reduced to [7.0 ms, 6.7 ms] for [2-ms LCD, μs/ns OLED/mLED/μLED displays], and it can be further shortened by half by doubling the frame rate to 240 fps. However, these displays are still much slower than the impulse driving CRT whose MPRT is approximately 1 ms.

An alternative method to shorten the MPRT is to globally dim the panel when the LC response is in transition and only illuminate the panel when the LC is ready. The ratio between the light emission time and the frame time is called the DR. In this way, the MPRT is shortened to

30 $${\mathrm{MPRT}} = 0.8 \times T_f \times \mathrm{DR}$$

Still taking the 60-fps display as an example, its MPRT can be dramatically shortened to 1.33 ms by applying a 10% DR, regardless of the LCDs or emissive displays. Recently, submillisecond MPRT has been achieved on LCDs by material development^83–85^, operation mode innovations^86^ and DR reductions^41,42^. However, the trade-off of using a 10% DR is decreased luminance. To achieve the same pixel luminance, the peak brightness of mLED backlight or the OLED (or μLED) pixels should be boosted by 10×. The lifetime degradation and efficiency droop effect should be taken into consideration.

## High dynamic range

HDR^87–90^ refers to the display standards aiming to faithfully reproduce natural scenes. Currently, a variety of HDR formats coexist^87^, such as the basic HDR10, the superb Dolby Vision, the broadcast-friendly Hybrid Log Gamma (HLG), and the rising Advanced HDR by Technicolor. An HDR display may support one or more HDR formats, but the hardware specs are more crucial to the final performance than the format adopted. In this section, we will discuss the necessities of the HDR display hardware^88,89^, namely, the high peak luminance, excellent dark state, high bit depth and wide colour gamut.

### Luminance

The human eye has a very wide dynamic range, covering an absolute specular highlight (10 000 cd/m^2^) to an extreme dark state (0.005 cd/m^2^)^88,90,91^. In contrast, the standard dynamic range display only offers a 100 cd/m^2^ peak luminance. As a manufacturer-friendly target, Ultra HD Premium defined the HDR luminance range as 0.05~ 1000 cd/m^2^ for LCDs and 0.0005~ 540 cd/m^2^ for OLED displays. This standard can be satisfied by all mLED/μLED/OLED display technologies. As a matter of choice, Dolby Vision is mastered at a 4000-cd/m^2^ peak luminance^88^. In 2020, Sharp’s 8 K LCD TV achieved over 10,000 cd/m^2^ by employing indium-gallium-zinc-oxide (IGZO) TFTs with an extremely low dark current and by boosting the backlight luminance^92^. The low optical efficiency-caused thermal issue can be partially addressed by local dimming technology. On the other hand, OLEDs suffer from efficiency roll-off^93^ and fast ageing^43^ at a high luminance, so they are more suitable for frequent-update devices. As a result, the mLED/μLED emissive displays demonstrate the best quality HDR preference for high luminance with high efficiency.

### Bit depth

With the expansion of the luminance range, 8 bits per colour is no longer sufficient to provide a smooth colour change. While 10 bits are applied in current HDR display systems, 12 bits per colour is highly desired to avoid banding artefacts according to the Barten model and the Perceptual Quantizer (PQ) curve^90,94^. Technically, at least 10 bits are required on the hardware if 2 bits are handled by dithering^95^. In conventional LCDs, the bit depth is limited by a large voltage swing and a slow grey-to-grey response time. Fortunately, the dual modulation units in local dimming LCDs share the burden equivalently so that the 12-bit PQ curve has been achieved^82,96^. In emissive displays, achieving 10-bit or 12-bit requires ultra-accurate current control in the PAM and ultra-short pulse generation in the PWM, leading to a high electronics cost. In 2018, JDC demonstrated a 10-bit µLED on a silicon backplane with PWM^65^. High bit depth is especially challenging when a low DR is applied to the PWM because it further reduces the shortest pulse width. Similar to the dual modulation in local dimming LCDs, hybrid driving^71^ could tackle the difficulties by combining PAM and PWM.

### Colour performance

Vivid colour is another critical requirement of HDR displays. There are various standards to evaluate the colour performance of a display panel, such as sRGB, NTSC, DCI-P3, and Rec. 2020^33–35^. The colour gamut coverage of the display is mainly defined by the central wavelength and full width at half maximum (FWHM) of the RGB emission spectrum. For example, Rec. 2020 is defined by red (630 nm), green (532 nm) and blue (467 nm) lasers^33,34^. In this section, we will report the colour gamut (x, y area coverage in CIE 1931) and colour shift of the mLED/μLED/OLED displays.

In 2017, SEL showed new materials to enable an OLED display with >101% (u’, v’) coverage, which corresponds to 91.8% (x, y) coverage in Rec. 2020^72^. Such a large colour gamut is achieved by material and device advancements: (1) Deep blue fluorescent and deep red phosphorescent OLED materials have been developed^14,66,72^, although further research is required to extend the device lifetime for commercial applications, and (2) the two metallic electrodes of the top emission OLED form a microcavity to significantly narrow the emission FWHM. The trade-offs are a compromised efficiency and a large angular colour shift. Therefore, proper OLED structure parameter optimizations^97^ and better cavity designs for mitigating colour shift^98^ are still needed.

Inorganic mLED/μLED inherently has a relatively narrow FWHM (18 ~ 30 nm)^99^, so the colour gamut mainly depends on the emission wavelength. Recently, 91.4% Rec. 2020 has been reported on the RGB-chip type^100^. A practical issue of PAM mLED/μLED displays is the central wavelength drift and the FWHM change with current^100^. As the current density increases, the central wavelength is blueshifted for the blue/green (InGaN) LEDs and redshifted for the red (AlGaInP) LEDs. As a result, the mixed white colour (D65) may not appear as white. This current-dependent colour shift can be minimized with the PWM. Inorganic mLEDs/μLEDs also have an angular-dependent colour shift, which results from the LED material difference and angular spectrum mismatch of the red and green/blue LEDs^70^. This problem can be solved by adding a black matrix to absorb the side emission to compromise the light extraction efficiency.

For the CC-type mLED/μLED emissive displays, the colour gamut is jointly determined by the blue LED chip and the green and red quantum dots. The narrow FWHM and high central wavelength tunability of QDs can theoretically enable >97% Rec. 2020^35^, and 93.1% has been experimentally demonstrated^101^. In this CC emissive display, additional attention should be paid to blue light leakage. The QDCF should be thick enough to effectively convert the blue light to red and green^44,102^, and an additional absorptive CF^44,45^ or DBR^46^ is needed to clean up the unconverted blue light and to minimize ambient excitations. As discussed above, the current-sensitive spectrum of inorganic mLEDs/μLEDs causes a colour shift on the blue subpixels under PAM so that PWM is still a preferred approach. In comparison, green and red quantum dots exhibit stable spectral emission profiles even though the wavelength and intensity of blue pumping light fluctuate. In addition, the colour shift may come from the angular emission profile mismatch between the blue LED and green/red quantum dots. To address this issue, scattering particles are added to the blue subpixels in the CC film to generate the same Lambertian angular profile as the green/red subpixels.

The colour gamut of mLED-LCD is dependent on the adopted CC material. From the Yttrium Aluminium Garnet (YAG) phosphor and K_2_SiF_6_ (KSF) phosphor to the QDs, the colour gamut is improved from ~50% and 70 ~ 80% to 80 ~ 90% Rec. 2020^103^. Different from the patterned CC film in emissive displays, the white backlight and absorptive CF in LCDs may introduce colour crosstalk and impair colour purity. Narrower band absorptive CFs could reduce crosstalk at the cost of a lower transmittance. In 2017, Chen et al. designed a bandpass filter in conjunction with green perovskite and red QDs to generate >95% Rec. 2020^104^. At large viewing angles, the gamma shift of the LCDs has been addressed by multidomain designs^36,37,39^ and compensation films^6,40^ to achieve an unnoticeable colour shift (<0.02).

**Fig. 8:**
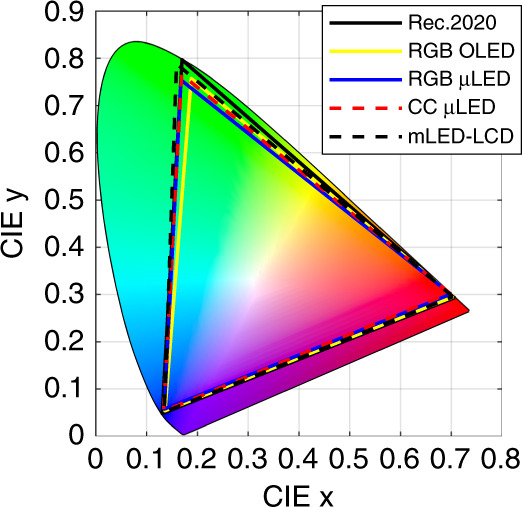
Chromaticity (x, y) of mLED/μLED/OLED displays in comparison with Rec. 2020

In summary, we compare the chromaticity diagram of mLED/μLED/OLED displays with Rec. 2020 in Fig. 8. A wide colour gamut (>90% Rec. 2020) can be obtained on all of them. It is a matter of choice to balance the colour gamut with the lifetime, colour shift, system efficiency, luminous efficacy and cost.

## Applications in novel scenarios

In this rapidly evolving information society, displays are ubiquitous. In this section, we take wearable electronics and vehicles as examples to illustrate new display trends, such as flexibility and transparency. The pros and cons of mLED-LCDs and mLED/μLED/OLED emissive displays will be analysed.

### Wearable displays

Wearable electronics, such as VR/AR headsets and smart wristbands, are believed to be next-generation information platforms. Common requirements for wearable displays are low power, light weight and high resolution density. Specifically, VR/AR near-eye displays demand a fast MPRT to reduce motion image blur, while smart wristbands prefer flexibility. We have already analysed the power consumption and MPRT issues. Here, we discuss the remaining issues.

**Fig. 9:**
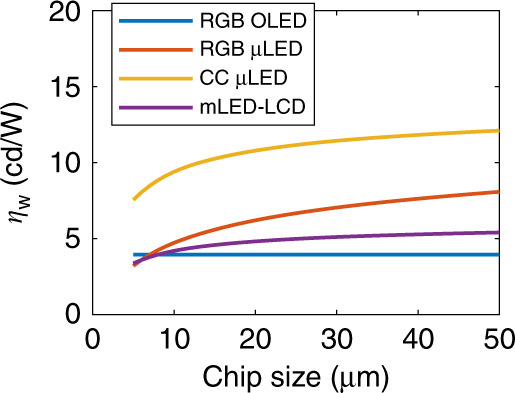
Chip size versus the on-axis power efficacy (*η*_*W*_) for the four specified display technologies

VR panels are operated in an immersed dark space so that the peak luminance of 150 ~ 200 cd/m^2^ should be adequate. This value corresponds to ~1000 cd/m^2^ instant luminance under a 15 ~ 20% *DR*. In Fig. 9, we plot the *η*_*W*_ of four different displays according to the peak EQE with different chip sizes. Ambient filters such as the CF on the CC μLED and the CP on the RGB-chip OLED/μLED are still laminated to clean up the ghost images. The efficiency ranks in the order of CC μLEDs, RGB-chip μLEDs, and mLED-LCDs to RGB-chip OLEDs when the LED chip size is over 7 μm. However, to eliminate the screen-door effect, an 100° field-of-view demands a 6K6K resolution, indicating 3000 ppi on a 2-inch panel and chip size < 5 μm. On such a small dimension, the CC μLED display is the most efficient, followed by the OLED display. On the other hand, foveation is an effective way to circumvent the high resolution/ppi hardware and software challenges^105^. This method releases 5× the burdens, embracing larger chips and LCDs^59,106^. Overall, a thin profile, high ppi, and high *η*_*W*_ make the performance of CC μLED emissive displays stand out, while the OLED display and mLED-LCD are mature and economic choices.

For AR devices, high luminance is critically important for the following reasons: (1) the displayed image overlays with environmental scenes so that the ACR matters. (2) In the space domain, a smaller panel means a higher luminance on the display if the same luminous flux is delivered to the human eye. The AR devices need much smaller panels than VR displays due to their increased optical system complexity. (3) In the time domain, a fast MPRT demands a high instant luminance. Numerically, we can use [AP · DR · Φ/Φ_LED_] to scale from the display luminance to the instant chip luminance, as discussed in the power consumption section. Because the lifetime of OLEDs is inversely related to their luminance^43^, inorganic LEDs have become the favoured choice. Currently, projection displays dominate the AR market. Liquid-Crystal-on-Silicon (LCoS) feature high luminance (>40,000 cd/m^2^)^107^ and high ppi (>4000)^108^, but the system is bulkier because it is a reflective display^24^. Pursuing a slimmer profile, laser scanning is an option, except that the optical efficiency remains relatively low. In recent years, some high luminance and high resolution density emissive microdisplays have been developed. In 2019, the BOE demonstrated a µOLED display with 5644 ppi and 3500-cd/m^2^ luminance^109^. On the other hand, μLED microdisplays have fulfilled all the requirements of a high luminance (>10,000,000 cd/m^2^)^23^, a high ppi (>5000)^110,111^, a fast MPRT, low power and a long lifetime. Moreover, the small chip size opens a new door for transparent displays^19,29^, which would tremendously simplify the optical configuration.

Smart wristbands have viewing conditions similar to smartphones. The unique technical challenge is flexibility. To fulfil this requirement, first, the light source should better be 2D arrayed, opening the door for emissive displays and mLED-LCDs. Second, the light source requires good off-axis performance. As discussed in the HDR section, the colour shift can be suppressed by various approaches. The main off-angle challenge comes from the quarter-wave plate in the CP. Therefore, CP-free small-aperture RGB-type and flexible QDCF^112^-laminated CC-type μLED emissive displays have the least physical limitations on flexibility and sunlight readability. On the other hand, the gamma shift on nonemissive LCDs has been well compensated^6,38–40^, and the integrated linear polarizer enhances the ACR. Researchers have developed organic TFTs for plastic substrates and flexible LCDs^113^. The so-called OLCDs have lower manufacturing costs and easier scalability for large panel sizes than do flexible OLED displays. Overall, OLEDs are the most mature flexible display technology, except their ACR is limited. New OLED materials with high EQE and long lifetimes are under active development^14^. The commercialization of flexible mLED-LCDs depends more on market strategies instead of technical challenges. Flexible μLED emissive displays are in the prototyping stage^19,29^. The CP-free small-aperture μLED is theoretically the best candidate.

### Vehicle displays

Typical vehicle displays for automobiles and spacecraft include central cluster panels and head-up display (HUD) units. For these applications, reliability and sunlight readability are critically important for driver safety. A wide working temperature is an additional demand on vehicle displays. Inorganic LEDs have the widest temperature range. OLED displays function well in freezing cold environments and age fast if heated^114,115^. LCDs respond slowly in cold weather, and the upper limit depends on the clearing temperature (*T*_c_). With extensive development efforts, LCs with *T*_c_ > 100 °C and 10-ms response times at −20 °C have been demonstrated^83^. Another drawback of LCDs is thermal management due to their low optical efficiency. Overall, mLED and μLED emissive displays show great advantages over OLED displays in luminance, lifetime and robustness in extreme environments.

In central clusters, a conventional LCD is the mainstream. With the alliance of the mLED BLU, a higher contrast ratio, lower power consumption, less heat generation and freeform factors are promising features to be realized. Micro-LED emissive displays may further enhance the HDR performance and power efficiency. Preferences with respect to the power efficiency can refer to the similar-pitch notebook in Fig. 6b.

The currently dominating HUDs in the market are LCD projection displays for the windshield or a postcard-size combiner^116^. There are several solutions to improve HUD quality: (1) Employing HDR panels to eliminate the postcard effect and gain higher peak brightness, where all mLED/μLED/OLED displays apply. (2) Enhancing the combiner reflectance of displays and smartly adjusting the ambient light transmission. An effective method is polarization modulation^117^. In this way, the display needs a polarizer at the output layer so that the optical efficiency of the CC μLED emissive display will be trimmed by half. Conceptually, transparent displays^19,29,30^ outperform projection displays with respect to the system complexity, optical efficiency, eyebox, field-of-view, etc. Technically, high transparency can be realized by utilizing either high conductivity transparent electrodes in PM displays^29^ or patterned transparent electrodes in AM displays^30^. Generally, a large aperture lays the foundation of high luminance in OLED transparent displays^30^, while they can be minified by employing μLEDs. To date, an ~ 70% transparency has been achieved on OLED^30^ and μLED^29^ displays. We believe the commercialization of transparent displays is coming soon.

## Conclusion

We have reviewed the recent progress and discussed the future prospects of emissive mLED/μLED/OLED displays and mLED backlit LCDs. All of these technologies support a fast MPRT, a high ppi, a high contrast ratio, a high bit depth, an excellent dark state, a wide colour gamut, a wide viewing angle, a wide operation temperature range and a flexible form factor. In realizing HDR, high peak brightness can be obtained on all mLED/μLED/OLED displays, except that mLED-LCDs require careful thermal management, and OLED displays experience a trade-off between lifetime and luminance. For transparent displays, all emissive mLED/μLED/OLED types work well. We especially evaluated the power efficiency and ACR of each technology. Among them, mLED-LCDs are comparably power efficient to circular-polarizer-laminated RGB-chip OLED displays. By removing the CP, the CC type and CP-free RGB-chip type mLED/μLED emissive displays are 3 ~ 4× more efficient. In addition, OLED displays and mLED-LCDs have advantages in terms of cost and technology maturity. We believe in the upcoming years OLED and mLED-LCD technologies will actively accompanying mainstream LCDs. In the not-too-distant future, mLED/μLED emissive displays will gradually move towards the central stage.

## Supplementary information

Supplementary information
