# Supplementary material for: Mini-LED, Micro-LED and OLED displays: present status and future perspectives
Source: Light Sci Appl. 2020 Jun 18;9:105. doi: 10.1038/s41377-020-0341-9 (PMC7303200; doi:10.1038/s41377-020-0341-9)
Supplement: Supplementary file 1 — Supplementary information [file 41377_2020_341_MOESM1_ESM.docx]

**Mini-LED, Micro-LED and OLED displays: Present status and future perspectives – Supplementary information**

*Yuge Huang^†^, En-Lin Hsiang^†^, Ming-Yang Deng^†^, and Shin-Tson Wu^*^*

*College of Optics and Photonics, University of Central Florida, Orlando, FL 32816, USA*

†These authors contributed equally to this work

*Corresponding author: [swu@creol.ucf.edu](mailto:swu@creol.ucf.edu)

This document provides supplementary information to “Mini-LED, Micro-LED and OLED displays: Present status and future perspectives”. We provide details in the *J*-*V_F_* characteristics, the evaluation of each display power efficiency. In particular, we disclose the LED chip characteristics and optical system parameters.

**Key Words:** Displays; mini-LED; micro-LED; organic light-emitting diode; power consumption; ambient contrast ratio

*J*-*V_F_* characteristics of μLED and OLED. The relationship between the current density of μLED (*J_μLED_*) and forward voltage (*V_F_*) can be described by the Shockley model^1,2^:

$J_{\mu LED}=J_{s}\left(e^{V_{F}/nV_{T}}-1\right).$ (S1)

Here, *J_s_* is the saturation current density, which is determined by the temperature, p-n junction diffusivity, diffusion length and carrier concentration. *V_T_* is the thermal voltage:

$V_{T}=\frac{k_{B}T}{q},$ (S2)

where *k_B_* is the Boltzmann constant (= 1.38 × 10^-23^ J/K) and *q* is the elementary charge (= 1.60 × 10^-19^ C). At room temperature (*T* = 300 K), *V_T_* = 25.9 mV. In Equation (S1), *n* is the ideality factor, which is equal to unity when bimolecular recombination is governing (corresponding to the high IQE region) and is close to 2 or even higher on multi quantum well LEDs^3^.

On the other hand, because of the small intrinsic charge density in organic materials, the current density of OLED (*J_OLED_*) is space charge limited^4–6^. According to the space-charge-limited-current (SCLC) model, the *J*-*V_F_* characteristic of OLED follows the Mott-Gurney law^7^:

$J_{OLED}=\frac{9}{8}\varepsilon_{0}\varepsilon_{r}\mu\frac{V_{F}^{2}}{d^{3}}.$ (S3)

Here, *ε_0_* is the vacuum permittivity (= 8.85 × 10^-12^ C/V·m), *ε_r_* is the relative permittivity of OLED material, and *d* is the distance between OLED electrodes (≈ 100 nm^8^). The free carrier mobility (*μ*) is a function of electric field (*E* = *V_F_*/*d*)^9^:

$\mu=\mu_{0}e^{0.89\beta\sqrt{\frac{V_{F}}{d}}},$ (S4)

where *μ*_0_ is the carrier mobility at zero electric field, and *β* is the Poole-Frenkel factor describing the strength of field-dependency:

$\beta=\frac{q}{k_{B}T}\sqrt{\frac{q}{\pi\varepsilon_{0}\varepsilon_{r}}}.$ (S5)

For estimation, *ε_r_* = 5~10 ^10,11^ corresponds to *β* = 0.013~0.0093 cm^1/2^/V^1/2^.

Generally, OLED requires higher operation voltage than μLED. As shown in Figure S1, OLED has a higher threshold voltage and a lower *J*-*V_F_* curve slope resulting from its much lower mobility (10^-10^ ~ 10^-2^ cm^2^/V·s, typically 10^-5^ cm^2^/V·s)^12^ than μLED (> 100 cm^2^/V·s)^2^.


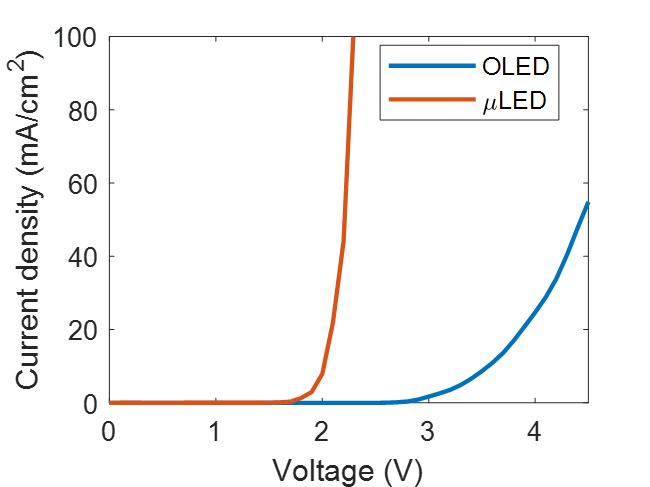


**Figure S1** *J*-*V_F_* characteristics of OLED and μLED (data from Refs. 13 and 14).

The *J*-*V_F_* curve slope of μLED and OLED can be derived from Equation (S1) and (S3):

$J_{\mu LED}^{'}\left(V_{F}\right)=\frac{J_{s}}{nV_{T}}e^{V_{F}/nV_{T}}\approx J_{\mu LED}\cdot\frac{1}{nV_{T}}.$ (S6)

$\begin{matrix} J_{OLED}^{'}\left(V_{F}\right)=\frac{9}{8}\varepsilon_{0}\varepsilon_{r}\frac{1}{d^{3}}\left[V_{F}^{2}\frac{\partial\mu}{\partial V_{F}}+\mu\frac{\partial V_{F}^{2}}{\partial V_{F}}\right] \\ =\frac{9}{8}\varepsilon_{0}\varepsilon_{r}\frac{1}{d^{3}}\left[\mu V_{F}^{2}\cdot\frac{0.89\beta}{2}\sqrt{\frac{1}{d\cdot V_{F}}}+2\mu V_{F}\right] \\ =J_{OLED}\cdot\left[\frac{0.89\beta}{2}\sqrt{\frac{1}{d\cdot V_{F}}}+\frac{2}{V_{F}}\right]. \end{matrix}$ (S7)

In Figure S2, we plot the *J*’/*J* of μLED and OLED as a function of forward voltage. Above the threshold voltage (> 2 V), μLED shows a higher *J*’/*J* than OLED, even with noticeable non-radiative recombination (*n* > 1). It means that μLED has a higher *J*-*V_F_* curve slope than OLED given the same current density (*J_μLED_* = *J_μOLED_*), which is consistent with the tendency shown in Figure S1.


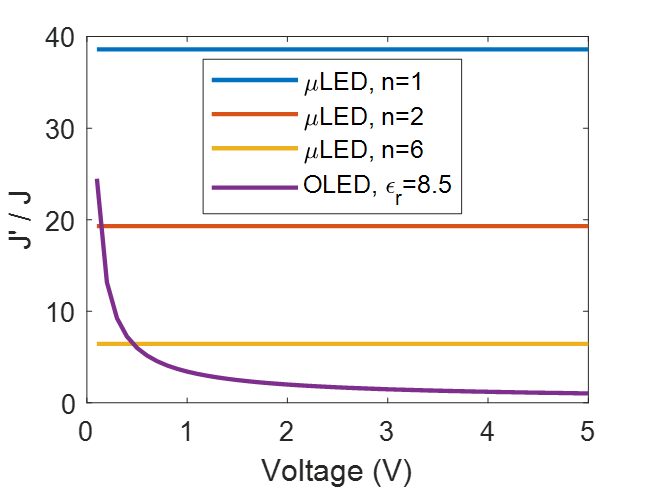


**Figure S2** *J*’/*J* of μLED and OLED as a function of forward voltage

Power efficiency evaluation. In the power consumption section in the main manuscript, we discussed the on-axis luminous power efficacy (*η_W_*) evaluation method. The data used in exemplary evaluation are summarized in Tables S1-S4. Tables S1 and S2 are for RGB-chip emissive displays with a mini-LED (mLED) chip set and an organic LED (OLED) chip set, respectively. Tables S3 and Tables S4 are for a color conversion mLED emissive display and a mLED backlit LCD, respectively.

Table S1 On-axis power efficacy of a RGB-chip mLED emissive display.

| Color | Red | Green | Blue |
| --- | --- | --- | --- |
| *K* (lm/W) | 260 | 652 | 77 |
| *E_ph_* (J) | 3.2×10^-19^ | 3.7×10^-19^ | 4.2×10^-19^ |
| *EQE_chip_* | 0.11 | 0.31 | 0.45 |
| *V_F_* (V) | 1.72 | 2.33 | 2.49 |
| *F* (lm/cd) | 4.67 | 3.67 | 3.67 |
| *T_CP_* | 0.42 | | |
| *r* | 0.270 | 0.616 | 0.114 |
| *η_RGB_* (cd/W) | 2.9 | 22.9 | 4.2 |
| *P*/*P_W_* | 0.63 | 0.18 | 0.19 |
| *η_RGB,W_* (cd/W) | 6.8 | | |

Table S2 On-axis power efficacy of a RGB-chip OLED emissive display.

| Color | Red | Green | Blue |
| --- | --- | --- | --- |
| *K* (lm/W) | 140 | 547 | 63 |
| *E_ph_* (J) | 3.1×10^-19^ | 3.7×10^-19^ | 4.3×10^-19^ |
| *EQE_chip_* | 0.27 | 0.24 | 0.10 |
| *V_F_* (V) | 3.98 | 3.40 | 3.20 |
| *F* (lm/cd) | 3.14 | 3.14 | 3.14 |
| *T_CP_* | 0.42 | | |
| *r* | 0.253 | 0.681 | 0.066 |
| *η_RGB_* (cd/W) | 2.5 | 12.1 | 0.7 |
| *P*/*P_W_* | 0.42 | 0.23 | 0.39 |
| *η_RGB,W_* (cd/W) | 3.9 | | |

Table S3 On-axis power efficacy of a color conversion mLED emissive display.

| Color | Red | Green | Blue |
| --- | --- | --- | --- |
| *K* (lm/W) | 207 | 561 | 77 |
| *E_ph_* (J) | 3.2×10^-19^ | 3.7×10^-19^ | 4.2×10^-19^ |
| *EQE_chip_* | - | - | 0.45 |
| *V_F_* (V) | - | - | 2.49 |
| *EQE_QDCF_*^15^ | 0.38 | 0.30 | 0.9 |
| *EQE_chip,B_* · *EQE_QDCF_* | 0.17 | 0.13 | 0.41 |
| *F* (lm/cd) | 3.14 | 3.14 | 3.14 |
| *T_CF_* | 0.82 | 0.91 | 0.72 |
| *r* | 0.293 | 0.594 | 0.113 |
| *η_CC_* (cd/W) | 7.4 | 20.6 | 7.5 |
| *P*/*P_W_* | 0.47 | 0.35 | 0.18 |
| *η_CC,W_* (cd/W) | 12.0 | | |

Table S4 On-axis power efficacy of a mLED backlit LCD.

| Color | Red | Green | Blue |
| --- | --- | --- | --- |
| *K* (lm/W) | 186 | 526 | 84 |
| *E_ph_* (J) | 3.1×10^-19^ | 3.7×10^-19^ | 4.3×10^-19^ |
| *EQE_chip_* | - | - | 0.5 |
| *V_F_* (V) | - | - | 2.8 |
| *EQE_QDEF_*^16^ | 0.73 | 0.73 | 1 |
| *EQE_chip,B_* · *EQE_QDEF_* | 0.37 | 0.37 | 0.5 |
| *T_BLU_* | 0.9 | | |
| *F* (lm/cd)^17^ | 0.96 | | |
| *T_LCD_* | 0.05 | | |
| *r* | 0.247 | 0.672 | 0.081 |
| *η_LCD_* (cd/W) | 2.2 | 7.4 | 1.9 |
| *P*/*P_W_* | 0.45 | 0.37 | 0.18 |
| *η_LCD,W_* (cd/W) | 4.1 | | |

Luminous reflectance of ambient light. The ambient light reflectance of a micro-LED (μLED) display is determined by the optical structure, such as the employment of circular polarizer (CP) and color filter (CF), and the emission aperture ratio of the LED chips. Figure S3 shows the luminous reflectance of D65 ambient light as a function of emissive aperture ratio. CP-laminated and CP-free RGB-chip emissive display as well as CF-laminated and CF-free color conversion (CC) emissive display designs are simulated. Significant reflection reduction by employing CP and CF is achieved.


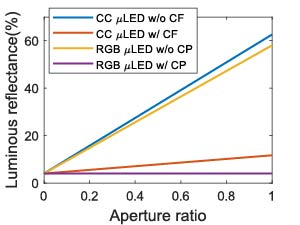


**Figure S3** Emission aperture ratio dependent ambient light luminous reflectance of the

Micro-LED external quantum efficiency (EQE). The *EQE_chip_* of μLEDs is size dependent. From the previous reports in μLED *EQE_chip_* measurement^18–20^, we summarize the state-of-the-art peak *EQE_chip_* as a function of R/G/B chip size in Figure S4. These *EQE_chip_* data are used in the simulation of Figure 6 in the main manuscript.


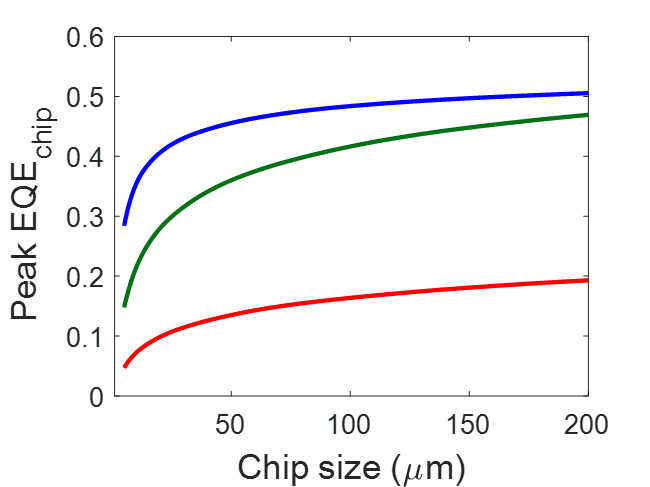


**Figure S4** Chip size dependent peak *EQE_chip_* of μLEDs. The R/G/B colors stand for the R/G/B μLED chips, respectively.

Supplementary information accompanies the manuscript on the Light: Science & Applications website ([http://www.nature.com/lsa](https://nam02.safelinks.protection.outlook.com/?url=http%3A%2F%2Fwww.nature.com%2Flsa&data=02%7C01%7Cswu%40creol.ucf.edu%7C2878766b304840d238b408d802c9a181%7Cbb932f15ef3842ba91fcf3c59d5dd1f1%7C0%7C1%7C637262412340626862&sdata=yqQGn4nf0nKnmgmNOHK2AdWwXqLckAfgb3gxj8YGeCA%3D&reserved=0))

References

1. Shockley, W. The theory of p‐n junctions in semiconductors and p‐n junction transistors. *Bell Syst. Tech. J.* **28**, 435–489 (1949).

2. Sedra, A. S. & Smith, K. C. Microelectronic circuits. (Oxford University Press, 2014).

3. Römer, F. & Witzigmann, B. Signature of the ideality factor in III-nitride multi quantum well light emitting diodes. *Opt. Quantum Electron.* **50**, 425 (2018).

4. Mark, P. & Helfrich, W. Space-charge-limited currents in organic crystals. *J. Appl. Phys.* **33**, 205–215 (1962).

5. Gaspar, D. J. & Polikarpov, E. OLED fundamentals: materials, devices, and processing of organic light-emitting diodes (Taylor & Francis Group, 2015).

6. Tsujimura, T. OLED display fundamentals and applications (John Wiley & Sons, 2017).

7. Mott, N. F. & Gurney, R. W. Electronic processes in ionic crystals (Oxford, Clarendon Press, 1940).

8. Liu, Y. *et al.* Locking excitons in two-dimensional emitting layers for efficient monochrome and white organic light-emitting diodes. *J. Mater. Chem. C* **7**, 8929–8937 (2019).

9. Murgatroyd, P. N. Theory of space-charge-limited current enhanced by Frenkel effect. *J. Phys. D. Appl. Phys.* **3**, 151–156 (1970).

10. Brütting, W., Berleb, S. & Mückl, A. G. Device physics of organic light-emitting diodes based on molecular materials. *Org. Electron.* **2**, 1–36 (2001).

11. Wang, F., Liu, S. & Zhang, C. The dielectric constant of materials effect the property of the OLED. *Microelectronics J.* **38**, 259–261 (2007).

12. Blakesley, J. C. *et al.* Towards reliable charge-mobility benchmark measurements for organic semiconductors. *Org. Electron.* **15**, 1263–1272 (2014).

13. Wong, M. S. *et al.* High efficiency of III-nitride micro-light-emitting diodes by sidewall passivation using atomic layer deposition. *Opt. Express* **26**, 21324–21331 (2018).

14. Salehi, A. *et al.* Realization of high-efficiency fluorescent organic light-emitting diodes with low driving voltage. *Nat. Commun.* **10**, 1–9 (2019).

15. Lee, E. *et al.* Quantum dot conversion layers through inkjet printing. *SID Symp. Dig. Tech. Pap.* **49**, 525–527 (2018).

16. Sadasivan, S., Bausemer, K., Corliss, S. & Pratt, R. Performance benchmarking of wide color gamut televisions and monitors. *SID Symp. Dig. Tech. Pap.* **47**, 333–335 (2016).

17. 3M. Vikuiti^TM^ dual brightness enhancement film (DBEF) (2008). at http://www.opticalfilters.co.uk/includes/downloads/3m/DBEF_E_DS_7516882.pdf

18. Ahmed, K. Micro LEDs efficiency targets for displays. *SID Symp. Dig. Tech. Pap.* **50**, 125–128 (2019).

19. Jung, T., Choi, J. H., Jang, S. H. & Han, S. J. Review of micro-light-emitting-diode technology for micro-display applications. *SID Symp. Dig. Tech. Pap.* **50**, 442–446 (2019).
